# Secular Mysticism: Entanglements of Science and Religion in Psychedelic Medicine

**DOI:** 10.1007/s11013-026-09988-x

**Published:** 2026-04-29

**Authors:** Aidan Seale-Feldman

**Affiliations:** University of Notre Dame, Notre Dame, United States

**Keywords:** Psychedelic medicine, Science, Religion, Psychiatry, Enchantment

## Abstract

Psychedelic medicine is a rapidly growing, billion-dollar industry poised to transform mental health care by incorporating spiritual experiences into clinical psychiatry. However, while the blending of psychiatry and mystical experience has long made this field unique, the blurred boundaries between science and spiritual practice have sparked increasing public debate. What does the entanglement of science and religion in psychedelic medicine reveal about the concerns, anxieties, and yearnings of our contemporary social and political moment? This article draws on an analysis of public discourse alongside ethnographic and qualitative research within a psychedelic church, a psychedelic-assisted therapy training program, and psychedelic science conferences in the United States. Through stories of the intertwining of science and religion, psychotherapy and mysticism, and attempts to distinguish between drugs, medicine, and sacraments in both clinical and non-clinical spaces, I argue that the mainstreaming of psychedelic medicine is not only shifting paradigms of mental health care but also creating new forms of secular mysticism in an age of disenchantment.

## Psychedelic Saints at the FDA

**Fig. 1:**
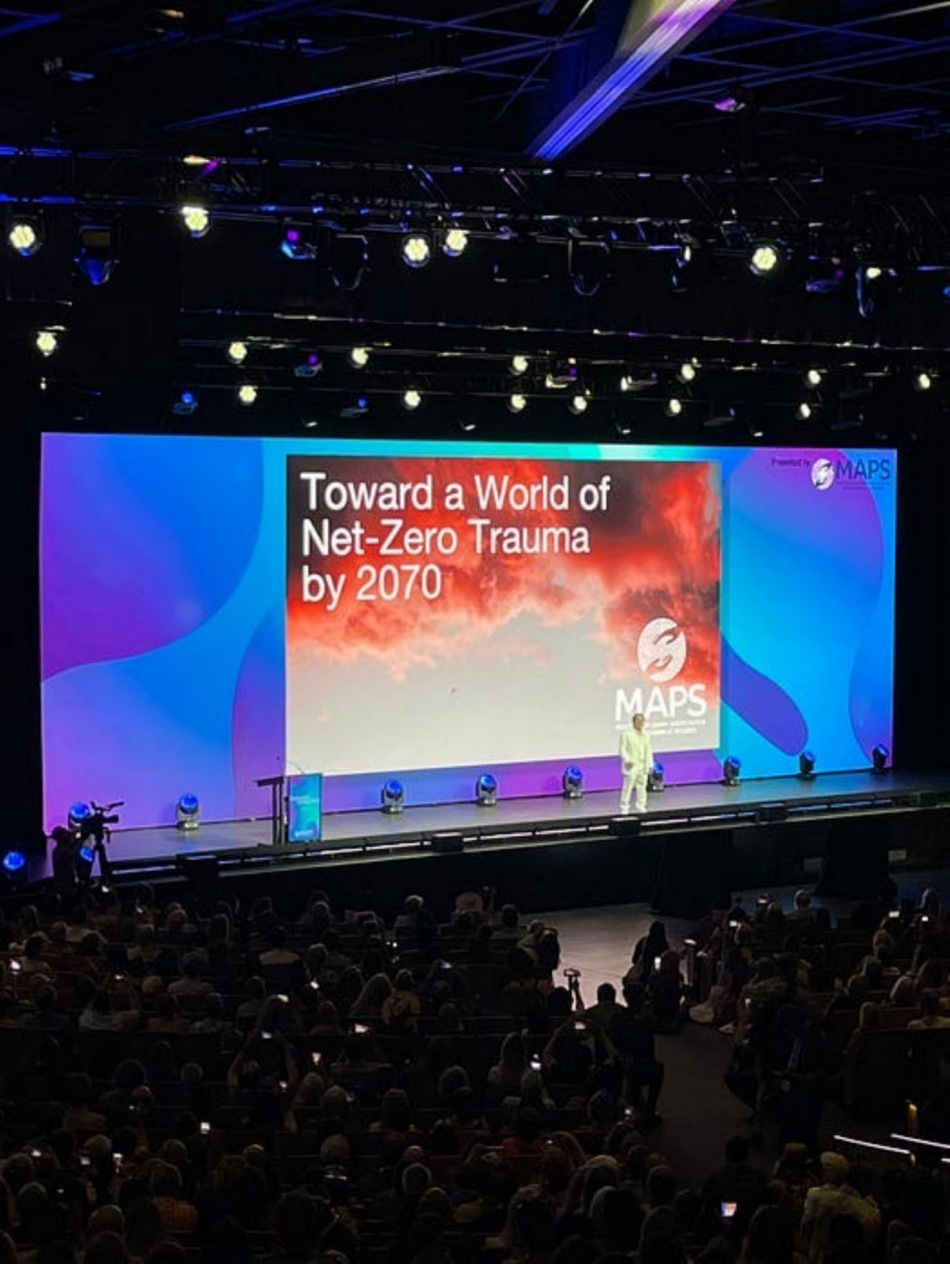
Rick Doblin addresses MAPS Psychedelic Science Conference Attendees in 2023. Photo by the Author

In June 2024, the US Food and Drug Administration (FDA) held a highly anticipated public meeting of an expert advisory panel to discuss the data from the Lykos Phase 3 clinical trial of 3,4-Methylenedioxymethamphetamine (MDMA) for the treatment of Post-Traumatic Stress Disorder (PTSD). The advisory panel was long awaited in the psychedelic community, many of whom were expecting FDA approval for the therapeutic use of MDMA, a Schedule I substance (known colloquially as “ecstasy”), in clinical settings.^1^ A year earlier, at the Multidisciplinary Association for Psychedelic Studies (MAPS) Psychedelic Science conference in Denver, over 16,000 people had gathered in the downtown convention center where they watched Rick Doblin, architect of MAPS/Lykos, deliver a speech about our psychedelic future. In Denver, Doblin paced the stage in a glowing white suit, as he described a vision of “a world of net zero trauma by 2070” thanks to globalized access to MDMA therapy. Lykos phase 3 clinical trial data seemed to show that MDMA, when administered under a controlled setting of therapeutic support, demonstrated increased efficacy in the treatment of PTSD compared to existing approaches. A contagious feeling of optimism around the inevitability of approval spread throughout the psychedelic community, who readied themselves for psychedelic-assisted therapy to finally emerge from the underground and into mainstream mental health care in the US (Fig. 1).

The FDA advisory panel, composed of a group of medical experts who lacked familiarity with the field of psychedelic science, initially focused their questions and concerns around issues of functional unblinding, the role of psychotherapy, and safety. For example, it is well known that psychedelic substances, including MDMA, are difficult if not impossible to blind in a clinical trial. The drug effects of these substances are so powerful and distinctly mind-altering that often the participant and anyone else in the room may be able to guess if they have been given the study drug as opposed to a placebo or an active control substance. When participants are able to figure out what they have been given, it becomes difficult to determine if the effect is due to the study drug itself or a placebo response. Beyond the challenges of blinding, the strong impact of set and setting (mindset and environment) on psychedelic experience has become a central problem for the FDA, which understands drugs to be substances that operate independently of the context in which they are taken. As one FDA regulator put it during a webinar on psychedelic clinical trials, “in contrast to most drugs we usually don’t pay any attention to where and how the patient is taking the drug.” Psychedelics, especially classical psychedelics like psilocybin and LSD, implode this model because of the major role that set and setting play in shaping the outcome of the experience (Dumit & Sanabria, 2022; Hartogsohn, 2017). As a result, in psychedelic medicine, the efficacy of the drug cannot be easily disentangled from the context, environment, and therapeutic modality in which it is delivered. Lastly, the FDA panel focused on questions of safety, particularly the risk of abuse potential of a substance that has long been celebrated as a club drug.

While these issues initially dominated the discussion of the advisory panel, during the public hearing that followed a new set of concerns surfaced. The people that gathered virtually to comment via online video streaming included military officers, Veterans, and military psychiatrists, participants in the clinical trial, people diagnosed with PTSD, researchers, and members of the psychedelic science community. While many of the public comments offered testimony in support of MDMA therapy for PTSD, a faction of psychedelic scholars and activists painted a radically different picture. Although some of the criticism addressed issues of misconduct within the clinical trial, specifically a case in which a study therapist was accused of sexually assaulting a participant, the thrust of their critique was focused on the dangerous presence of spirituality in the Lykos organization, its treatment model, and the wider field of psychedelic science.

One high-profile activist said “This committee has been misled by Lykos.” Showing a provocative slide with a painting by psychedelic artist Alex Grey titled “Stanislav Grof M.D., Cartographer of Consciousness” from Grey’s “Psychedelic Saints” series, the researcher continued, “[Rick] Doblin admits that Lykos’ entire approach is based on Grof’s spiritual teachings, and that the essence of the MDMA treatment approach is a Grofian ‘death–rebirth’ process.” In the painting, the pioneering psychedelic psychiatrist and inventor of “breathwork,” Stanislav Grof, is depicted with a third eye, painting a birth sequence while surrounded by small icons of Sigmund Freud, Carl Jung, Otto Rank, and his wife and collaborator, Cristina Grof. At the top of Grof’s head, a phoenix erupts into divine light.^2^ Another speaker added, “It is Rick Doblin’s goal to have a global spiritualized society by 2070. Controversial New Age psychiatrist Stan Grof comes up 14 times in the therapy manual. He believes all psychopathology is rooted in traumas in the birth canal.” A third speaker, who introduced himself as a psychonaut^3^ and whistleblower, said “I feel compelled to echo concerns warning the public of what is an openly known secret in the psychedelic industry. That, in the words of Dr. Doblin, his long-term desire is a ‘spiritualized humanity,’ politically advancing this mission using the reputation of Science. The study has functioned as that first politically savvy Trojan Horse of that mission.” A fourth speaker concluded, “I submit that Lykos is a therapy cult that uses the application under review to further mystical and utopian goals.”

The attack against Lykos that was launched during the FDA’s public hearing was surprising to many members of the psychedelic community. And while it may have influenced the FDA’s ultimate decision two months later to reject Lykos’ application for MDMA to treat PTSD, it is also significant for what such critiques reveal about the changing role of mysticism and spirituality in the United States. The public debate surrounding the presence of spirituality in the clinical trial and MDMA-assisted psychotherapy more generally illuminated two long-standing and conflicting understandings of psychedelics. From a scientific perspective, psychedelic substances are understood as psychiatric medication that operates on neurochemical systems in the brain, while from a religious perspective, they are sacred and spiritual sacraments, plant teachers, and conduits of the divine. The growing tension between these two sides of psychedelics in the new “psychedelic renaissance” reflects both a desire for and ambivalence around spirituality in secular American culture.

In the literature and in practice, psychoactive substances that bring about “mystical experiences” are variously referred to as “psychedelics” and “entheogens,” and the co-existence of these terms speaks to their ambiguous position along the border between the secular and the sacred. The term “psychedelic,” defined as “mind-manifesting,” was first coined by the Canadian psychiatrist Humphrey Osmond in 1957 to describe the way that substances like mescaline could reveal aspects of the mind not normally available to consciousness (Tanne, 2004). For Osmond and other psychiatrists during the first wave of psychedelic research, psychedelics were tools that could be used to model psychosis (Dyck, 2008; Langlitz, 2013). Today neuroscientists continue to study the effects of psychedelics on the brain to gain new insights into the functioning of neurocognitive systems and consciousness itself (Carhart-Harris & Friston, 2019).

On the other hand, the term “entheogen” is drawn from the ancient Greek *entheos,* meaning “god within,” and *genesthai,* meaning “to come into being.” The term “entheogen” was introduced as an alternative to “psychedelic” and “hallucinogen” in 1979 by Carl Ruck and his colleagues in an effort to emphasize the qualities of transcendence and communion (as opposed to mere hallucination) that often accompany the ingestion of such substances (Winkelman & Hoffman, 2015). The use of “entheogen” thus implies that “psychedelic” substances reveal the presence of the sacred. As Godlaski writes, entheogens “produce a state in which a person experiences a sense of being inspired/transported beyond…in a way identified as distinctly religious or spiritual” (Godlaski, 2011, 1217).

Since the mid-1950s, there have been stark divisions between the sacred and the scientific dimensions of psychedelics (Dyck, 2008, 79). As historian Erica Dyck notes, while early psychedelic psychiatrists were in dialogue with figures such as Al Hubbard and Aldous Huxley who sought to explore LSD as a spiritual aid, ultimately, they were reluctant to accept a nonmedical view even as psychedelics were being conceptualized as spiritual enhancers in broader popular culture. In the contemporary entanglement between the two sides of psychedelics, the sacred and the scientific, a new iteration of this history is playing out in which the scientific domain of psychedelics has become increasingly permeable to the spiritual, and vice versa.^4^

Today, psychedelic medicine is a rapidly growing, billion-dollar industry that seems poised to transform mental health care by incorporating mystical experiences into clinical psychiatry. However, while the blending of psychiatry and mysticism has long made this field unique, the blurred boundaries between science and spiritual experience, secular and sacred, have sparked increasing public debate. What does the entanglement of science and religion in psychedelic medicine reveal about the concerns, anxieties, and yearnings of our contemporary social and political moment? Many participants in the new era of psychedelic research are challenged by the mystical experiences that such substances may elicit, which shake up secular worldviews, reconfigure biomedical approaches, and continually blur the boundaries between science and religion.

The secular, Talal Asad argues, is “a concept that brings together certain behaviors, knowledges, and sensibilities in modern life” (2003, 25); it works through oppositions such as belief vs. knowledge, natural vs. supernatural, sacred vs. profane and places “religion” in the sphere of the irrational.^5^ Charles Taylor (2007) has argued that the Western world is living in a secular age, a shared condition that has shaped possibilities for lived experience and created a new “buffered” form of subjectivity that is invulnerable to spirits, gods, and the supernatural. Taylor defines secularity not as secularism or the waning of religion, but as “a move from a society where belief in God is unchallenged…to one in which it is understood to be one option among others” (Taylor, 2007, 3). In this way, secularity is “a matter of the whole context of understanding in which our moral, spiritual or religious experience and search takes place” (Taylor, 2007, 3) for the religious and non-religious alike. In a secular age of “buffered subjects” (Taylor, 2007) who have liberated themselves from the powers of gods and spirits through rational empirical knowledge and the mastery of science, the phenomenology of psychedelic experience punctures the boundaries of a disenchanted world (Weber, 2004).

This article is based on 18 months of ethnographic research on the incorporation of mystical experience into the therapeutic practices of secular American culture, via the mainstreaming of psychedelic medicine. Methods involved multi-sited, ethnographic fieldwork with entheogenic churches, a psychedelic-therapy training program, and in psychedelic science conferences in the US, alongside 45 semi-structured interviews and an analysis of public media that includes news articles, social media posts, and popular publications. Following Asad and Taylor’s writings on secularity as an ethos and context of understanding that shapes relationships to transcendence, I define “secular American culture” as a powerful and pervasive worldview in which scientific and materialist explanations are valued over those offered by religion, and belief in God is seen as one option among many. Regardless of personal belief, the sociocultural context of secularity in the US shapes how people relate to the presence of mystical experience in psychedelic medicine. The majority of the people I spoke with across all field sites described themselves as “spiritual but not religious,” a growing category of people in the US that reject religion yet yearn for spiritual experiences of transcendence and self-transformation that have been banished from secular life. Such spirituality is a *mode of secular seeking* that is produced by the secular and its limits, that is, “in the gaps of secular, materialist epistemology” where “secular materialism overspills its bounds” (Farman, 2019, 59). Throughout this article I use the terms “religion” and “spirituality” in the Western liberal sense (Asad, 1993, 112) to refer to a domain in which supernatural forces, gods, spirits, and experiences of transcendence are possible, as opposed to a disenchanted scientific worldview that treats such experiences as irrational. Through stories of the intertwining of science and religion, psychotherapy and mysticism, and attempts to transform drugs into sacraments in both clinical and non-clinical spaces, I argue that the mainstreaming of psychedelic medicine is not only shifting paradigms of mental health care but also creating new forms of secular mysticism in an age of disenchantment.

## Psychedelics and the Re-Enchantment of the World

“Psychedelic” substances have been used in a variety of contexts and ways, ranging from religious sacraments and ritual healing medicines to psychiatric treatments and covert weapons of psychological warfare.^6^ In the United States, the popular use of psychedelics (and LSD in particular) has a distinct history that is closely connected to the rise of the countercultural movement in the 1960s. During this time of social and political crisis, users of psychedelics celebrated them as tools for the expansion of consciousness and the enhancement of creativity as well as spiritual evolution, the deconstruction of social roles, and the creation of a utopian future (Lee & Shlain, 1994). While today this culture of use is being augmented by new processes of medicalization, the legacy of the psychedelic counterculture continues to inform modes of engagement with psychedelics in the US.

Prior to the development of psychedelic psychiatry and the counterculture that followed, substances such as psilocybin, ayahuasca, and peyote had long been used in indigenous healing traditions as well as religious rituals throughout the world, particularly in North and South America, and continue to be an important part of these cultural practices (Dobkin de Rios, 1984; LaBarre, 1960, 1970; Mooney, 1896). Among the Mazatec people of Mexico, psilocybin mushrooms, known as *los niños* or “holy children,” are used to heal and cure the sick by imparting their wisdom to the healer during ritual healing sessions. As the famed Mazatec healer María Sabina explained in her life history, “the mushrooms are saints; they give me wisdom” (Estrada, 2003, 22) and “the sick arrived looking pale, but the mushrooms told me what the remedy was. They advised me what to do to cure them” (Estrada, 2003, 25).

In syncretic ayahuasca churches such as Santo Daime that first emerged in Brazil in the 1930s, Catholic religious practices, Spiritism, and Afro-Brazilian Umbanda are combined with “psychedelic sacraments” to facilitate transformative experiences of communion with what William Barnard describes as “the divine source of it all” (Barnard, 2022, 54). Through the ritual use of the *daime* (ayahuasca), the Daimista is drawn into a world of “aliveness” and “moreness,” that is “impregnated with a sense of portentous meaningfulness and significance” (Barnard, 2022, 68). As Barnard has pointed out, depending on the set and setting, “psychedelics are thought to generate experiences that are inherently valuable, true, and spiritually transformative” (Barnard, 2022, 54). Alongside entheogenic churches, ayahuasca healing centers and retreats grounded in Indigenous Amazonian traditions have become increasingly popular destinations for secular moderns seeking meaning, healing, self-transformation, and spiritual experience (Dupuis, 2022; Gearin, 2024; Labate & Cavnar, 2014). As an Australian ayahuasca drinker explained to Alex Gearin in his study of the social and cultural shaping of neoshamanic ayahuasca experience, “It was life changing….Ayahuasca brew became…my contact with spirituality” (2024, 159).^7^

The spiritual qualities of psychedelics have also been noted in numerous publications such as *Psychoactive Sacramentals,* which contains chapters by Stanislav Grof and Albert Hofmann, inventor of LSD, among other psychedelic luminaries (Roberts, 2001). Here Grof shares the story of his first psychedelic experience, which took place in a clinical setting, where he encountered what he describes as “Cosmic Consciousness” (2001, 31) and was subsequently transformed from an atheist into a seeker of spirituality and mysticism. Grof critiques the scientific, materialist perspective and psychiatry in particular for its rejection of the spiritual and sacred dimensions of existence, arguing that psychiatry’s inability to distinguish between psychosis and spiritual emergence has resulted in the pathologization of “the entire spiritual history of humanity” (2001, 35). In a subsequent chapter titled “LSD as a Spiritual Aid,” the Swiss chemist Albert Hoffman shares a similar story about the influence of LSD on his worldview and notes the role of invisible forces in his discovery of the compound. “I did not look for it, it came to me,” he writes. “This means to me that a higher authority thought it was necessary now to provide mankind with an additional pharmacological aid for spiritual growth” (Hofmann, 2001, 121). In contrast to what Langlitz (2013, 241) describes as the “mystic materialism” of hallucinogen researchers who are “less committed to a neurotheology of mystical experiences than to a biomysticism in awe of life itself,” in the conversion stories of Grof and Hoffman the phenomenology of the psychedelic experience repeatedly challenges the secular modern worldview of the atheist-scientist, forcing him to awaken to a mystical nature of reality that had previously been disenchanted.

The idea of disenchantment was first introduced by Max Weber in his famous 1919 lecture, “Science as Vocation.” The lecture was delivered at the University of Munich, to an audience of students seeking guidance, both moral and existential, and reassurance of the relevance of their academic studies. Yet instead of reassuring them, Weber not only outlined the limitations of science as a tool for offering answers to existential questions, but argued that science had brought about a kind of “existential emptiness” (Harrington, 1996, xi). “We are not ruled by mysterious, unpredictable forces, but on the contrary, we can in principle control everything by means of calculation,” wrote Weber. “That in turn means the disenchantment of the world” (Weber, 2004, 13). For Weber, disenchantment via scientific progress had not only propelled modern Europe into an age of alienation from the divine, but also from *meaning* itself.

This strand of German thought has had a far-reaching impact on understandings of modernity and the contemporary condition. For example, concerns with disenchantment appear again in the philosopher Charles Taylor’s work *A Secular Age.* Here Taylor tells a story about the shift from a society where belief in God was unopposed to a society where such belief is one option among many. Taylor is specifically interested in tracking the ramifications of this shift, and how modern secularity has impacted lived experience and ways of being-in-the-world. Primary among the effects of this transformation, Taylor argues, has been the creation of “the buffered subject.” As Taylor writes, “living in a disenchanted world, the buffered self is no longer open, vulnerable to a world of spirits and forces which cross the boundary of the mind…” (Taylor, 2007, 300). Taylor argues that the “buffered identity” can ultimately lead to a feeling of lack of meaning and purpose. “Our age suffers from a loss of meaning,” he writes, echoing Weber’s early critiques of disenchantment. “This malaise is specific to a buffered identity, whose very invulnerability opens it to the danger that not just evil spirits, cosmic forces or gods won’t ‘get to’ it, but that nothing significant will stand out for it” (Taylor, 2007, 303). According to Taylor, such “malaises of modernity” have inspired increasing numbers of people to seek alternative sources of spirituality and transcendence to fill the abyss of meaning in their lives.

However, claims that we are living in a disenchanted world have not gone without critique. For example, Jason Josephson-Storm (2017) has argued that while narratives of disenchantment may signal a conflict between modernity and magic, they do not prove the latter’s disappearance. Josephson-Storm offers numerous examples that complicate any straightforward account that rational, scientific knowledge has banished magic and spirits from the Euro-American world.^8^ In doing so, he shows how the diagnosis of a disenchanted world appeared simultaneously alongside an occult revival. At the same time, one can read the popularity of the occult during the emergence of the disenchantment narrative as further indication of an underlying “secular malaise” (Taylor, 2007) and spiritual yearning that had to be hidden away from secular life and its scientific worldview.

Secular malaise is likely to be widespread in the United States, where according to the 2024 Pew Research Center’s National Public Opinion Reference Surveys, 28% of the US population describe themselves as religious “Nones,” a group of religiously unaffiliated adults that include atheists, agnostics, secular humanists, and “nothing in particular,” as well as the “spiritual but not religious” (Pew Research Center, 2024). Researchers who have tracked the steady rise of the religiously unaffiliated argue that there are multiple factors driving this group’s expansion, including processes of secularization as well as a broader shift in American society from the centrality of community to a withdrawal from social life (Burge 2021; Putnam 2000). While almost 1 in 3 Americans now describe themselves as religious “Nones,” according to Fuller and Parsons (2018), millions of Americans with no connection to organized religion nevertheless identify themselves as religious/spiritual. According to a recent survey, “most ‘Nones’ believe there are limits to what science can do,” with 56% believing “there are some things science can’t possibly explain” (Pew Research Center, 2024, 14).

Theories of disenchantment, “malaises of modernity,” and the emergence of a secular age provide an important framework that can help to understand the complex entanglements of science and religion in psychedelic medicine today, as well as the broader problem of mystical experience in secular American culture. A growing dissatisfaction with the materialist worldview is evident in the United States, where there is a strong undercurrent of desire for spiritual healing and forms of knowledge that fall outside the scope of secular humanism. Such desire is reflected not only in the growth of the “spiritual but not religious” population, but also in the popularization of entheogenic churches, “natural” medicines, therapies like Internal Family Systems (IFS) that exorcise “unattached burdens” (Falconer, 2023, 19), and the “spiritual turn” in the art world (Heartney, 2025), as well as in intellectual movements such as the “ontological turn” that seeks to “take seriously” the possibility of spirits and the agency of non-human entities (Holbraad & Pedersen, 2017). Controversies over the boundaries between science and religion in psychedelic medicine play out against this backdrop of secular mysticism, in which people struggle to find ways to integrate spiritual experience into secular lives and spaces.

## Religion in the Lab

From the Good Friday Experiment in 1962 to the publication of Rolland Griffith’s groundbreaking study in 2006 demonstrating that “psilocybin can occasion mystical-type experiences having substantial and sustained personal meaning and spiritual significance,” psychedelic science has been entangled with questions of religion and spirituality. The Good Friday Experiment, conducted by Walter Pahnke, then graduate student in theology under the guidance of Timothy Leary at Harvard, involved dosing twenty Protestant divinity students with either psilocybin capsules or a placebo in the basement of Boston University’s Marsh Chapel while they listened to a sermon piped in from the main chapel above. The aim of the study was to compare the mystical experiences induced by psychedelics such as psilocybin, LSD, and mescaline to the experiences of religious mystics (Pahnke, 1963). Pahnke’s study led to the development of a questionnaire to measure the mystical experiences of his subjects, which used categories similar to those outlined by William James and Walter Stace (Doblin, 1991; Stace, 1960; James, 1902). Today, versions of the Mystical Experience Questionnaire (MEQ), such as the revised MEQ30, continue to be used as a standardized tool in psychedelic clinical trials to measure the presence and intensity of mystical-type experiences such as transcendence of time and space, ineffability, noesis, unity, and sacredness occasioned by psychedelics (Barrett et al., 2015).

Griffiths’ 2006 study on psilocybin and mystical experience marked the arrival of a new era in psychedelic science. In this work, he evaluated the long-term psychological effects of a high dose of psilocybin compared to a control compound when administered to hallucinogen-naïve adults under supportive and comfortable conditions. The double-blind study found that the mystical experiences occasioned by psilocybin shared marked similarities to classic mystical experiences reported in religious literature, and that 67% of participants rated their experience with psilocybin to be “either the single most meaningful experience of his or her life or among the top 5 most meaningful experiences of his or her life” (Griffiths et al., 2006, 276).

In 2016 Griffiths and his team at Johns Hopkins published the results of a subsequent randomized double-blind trial on the impact of psilocybin on depression and anxiety in patients with life-threatening cancer. In this study, 51 cancer patients with life-threatening diagnoses and symptoms of depression and anxiety were administered both a high and low dose of psilocybin. The researchers found that “high-dose psilocybin produced large decreases in clinician- and self-rated measures of depressed mood and anxiety along with increases in quality of life, life meaning, and optimism, and decreases in death anxiety” (Griffiths et al., 2016, 1181). The study concluded that “mystical-type psilocybin experience on session day mediated the effect of psilocybin dose on therapeutic outcomes” (Griffiths et al., 2016, 1181). Griffiths et al.’s finding that the therapeutic efficacy of psilocybin was correlated with the intensity of mystical experience established the critically important role of the presence of mysticism and spiritual experience in the new psychedelic psychiatry.^9^

In 2023, Roland Griffiths died of cancer. Soon after, the *New York Times Magazine* published an article on his work titled “The Psychedelic Evangelist.” The article questioned the rigor of Griffiths’ research and accused him of incorporating aspects of religion and spirituality into his clinical trials in ways that may have influenced their outcomes (Borrell, 2024). His critics noted that Griffiths’ lab contained spiritual books and a statue of the Buddha and said that study drugs were administered to participants in a ritual chalice. In the words of Matthew Johnson, one of Griffiths’ former colleagues, Griffiths was acting like a “spiritual guru” when he should have been in the role of the scientist. In an earlier academic article, Johnson had warned of what he called “the guru effect” in psychedelic medicine, writing that, “It can be challenging to be associated with what might be one of the most meaningful experiences in a person’s life. The scientist or clinician might, perhaps without explicit awareness, fall into the trap of playing guru or priest…” (Johnson, 2021, 580). To his critics, Griffiths’ own beliefs in the spiritual value of psychedelics had compromised the integrity of his work, thus leading him to abandon the central “epistemic virtue” of scientific research: objectivity (Daston & Galison, 2007).

Such public critiques of Griffiths’ research illuminate the challenge of secular mysticism, in which psychedelics in the lab must be protected from any external suggestion of spirituality, despite their common ability to generate spiritual experiences. Fears of losing scientific credibility by publicly pursuing interest in psychedelics’ spiritual qualities have long haunted clinical researchers in the field. As Dyck notes in her history of the early years of psychedelic psychiatry in 1950s, although leading researcher Abram Hoffer was fascinated by LSD’s spiritual applications, he “was not prepared to fully endorse a spiritual model for explaining the effects of LSD, which he felt dissolved any remaining medicoscientific credibility from the original studies” (2008, 99) and threatened his “professional integrity” (2008, 100).

Thus far this article has focused on an analysis of public discourse about the place of spirituality and religion in psychedelic science. In what follows, we turn to a set of ethnographic descriptions based on long-term fieldwork in a psychedelic-therapy training program and a psychedelic church where the “spiritual but not religious” use psychedelic sacraments with the hope of having a direct experience of the divine in this lifetime.

## Making Biomedicine Sacred

The ketamine apprenticeship training of the Vision Quest psychedelic-assisted therapy training program was held over four days in a luxe and relaxing retreat center.^10^ Like many “experiential” trainings in the field of psychedelic-assisted therapy, the retreat involved a ketamine practicum in which participants would both take ketamine and accompany or “sit for” someone else in a carefully constructed group setting. While optional, the ketamine practicum was the capstone of a 6-month training program in psychedelic-assisted therapy, run by a leading organization in the field. As part of my fieldwork, I conducted participant observation in the training program, interviewed trainees, attended the ketamine training retreat, and received a certification in psilocybin facilitation.

**Fig. 2:**
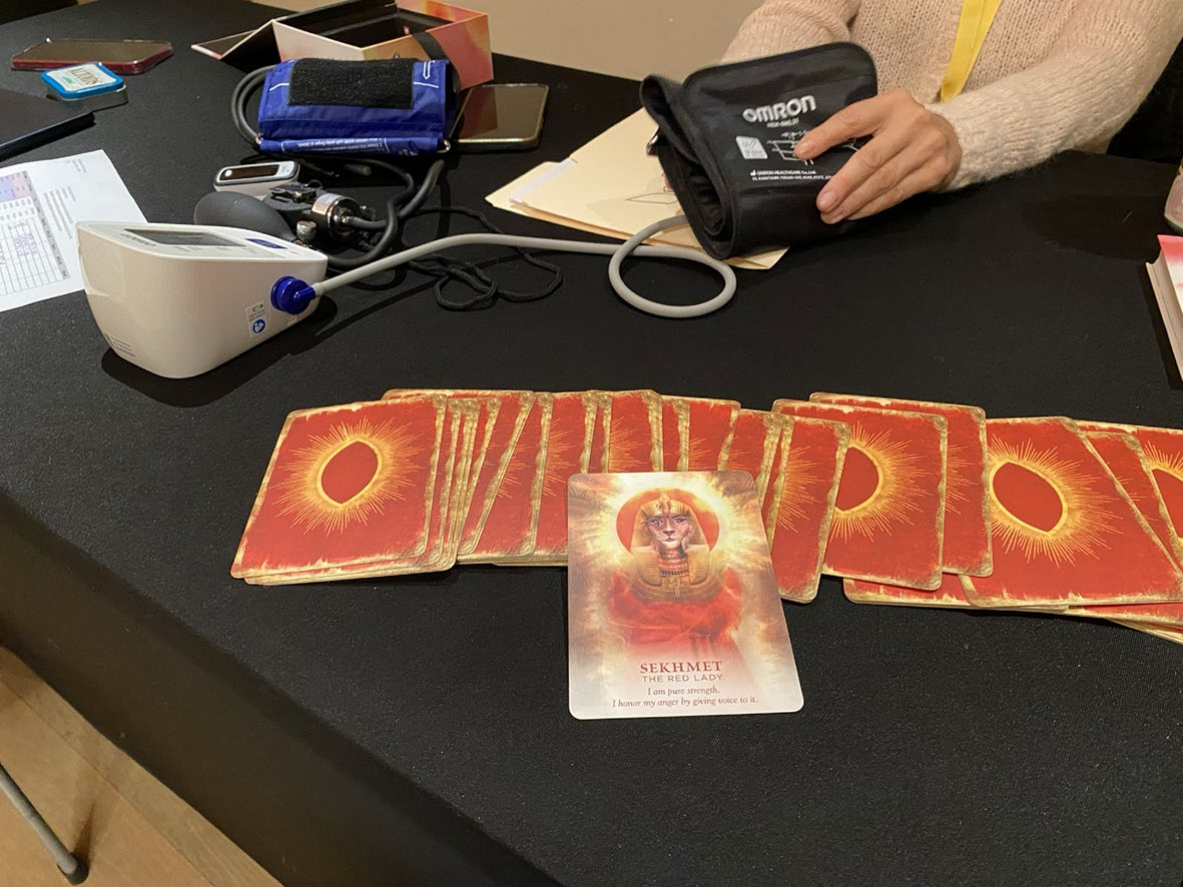
Tarot cards at the medical intake table. Photo by the Author

When I arrive at the retreat, my first stop is to the medical check-in area to meet with the ketamine provider for my group. My doctor is a stylish man, wearing a small scarf around his neck and a stethoscope. As the nurse takes my blood pressure, I notice two decks of Tarot cards on the table among the medical equipment, one “archetypal” and one “divine feminine.” I ask about the decks; they belong to the nurse. She says she loves archetypes and asks if I wanted to draw a card. I stay and talk to the nurse for a while. I tell her about my research and about my interest in how spirituality appears across different psychedelic spaces. She says she really loves that element of psychedelics, and that she dislikes the clinical context more and more. She says she thinks these substances just aren’t really well suited for the clinic, and that that’s why the FDA application failed, because you can’t blind these medicines. She says she prefers the underground, group work, that it’s not exactly therapy but its therapeutic (Fig. 2).

The next day in the morning lecture on ketamine, a psychiatrist opens his presentation to the lecture hall of 150 students with a quote from Stanislav Grof describing ketamine as the “strangest most extraordinary substance.” The psychiatrist begins by telling us that the “Western medical paradigm is a dogma” and that “there are limitations to the rational scientific approach.” He says it’s “an exciting time to be living in,” whether you are a doctor or a shaman, before launching into a lecture on the “American mental health crisis” and the failure of existing pharmaceutical treatments to help people with treatment-resistant depression. He shows a slide with a ketamine vial and an image of the molecule. “Admire the beautiful ketamine vial” he says. “Some of these vials are sitting in your pod rooms.” He then honors the Mazatec healer María Sabina, “for her inspiration and healing that she brought to this,” as well as Albert Hoffmann and Calvin Stevens, inventor of ketamine, before turning to pharmacokinetics. He tells us the story of how the Johnson & Johnson company patented half of the ketamine molecule and called it “esketamine.” “S stands for sinister” he says. “That’s the biomedical model” (i.e., biomedicine is synonymous with profit-driven motives over healing).

Given the familiar refrain about crisis, disenchantment, and the limits of the scientific worldview it is worth taking a brief foray into prior historical periods when similar concerns have come to a head to understand the present with more clarity. In her study of “holistic science” in German culture between WWI and WWII, Anne Harrington tracks the emergence of a new science of Wholeness that appeared both as a response to critiques of science as overly mechanistic, and as solution to a series of social and political crises understood to be tied to modernity, positivism, materialism, and the role of the machine in society (Harrington, 1996, xx). The holistic movement in the life and mind sciences gave rise to fields such as Gestalt psychology, psychosomatic medicine, and the vitalist concept of *umwelt*, in which the organism and its environment are envisioned as an integrated system.

While the story Harrington tells is specific to interwar Germany, she also points out important parallels in the United States. In the countercultural 1960s, a new generation of hippies and Leftists similarly critiqued both science and the role of capitalism in society. It was during this era that holistic and New Age therapies, including the work of Stanislav Grof, first became popular as more integrated and relational solutions to the ills of society (Harrington, 1996, 220). Today we are again living through a radical shift in attitudes toward science, health, and medicine in the United States and the popularization of psychedelic medicine fits into this new era. As US Secretary of Health and Human Services RFK Jr. wrote in a 2024 post on X, “FDA’s war on public health is about to end. This includes its aggressive suppression of psychedelics, peptides, stem cells, raw milk, hyperbaric therapies, chelating compounds, ivermectin, hydroxychloroquine, vitamins, clean foods, sunshine, exercise, nutraceuticals, and anything else that advances human health that can’t be patented by Pharma.” Such critiques of Big Pharma, biomedicine, and the limits of a secular, materialist approach to affliction were palpable throughout the ketamine training, from the content of lectures to the administration of the ketamine itself.

**Fig. 3:**
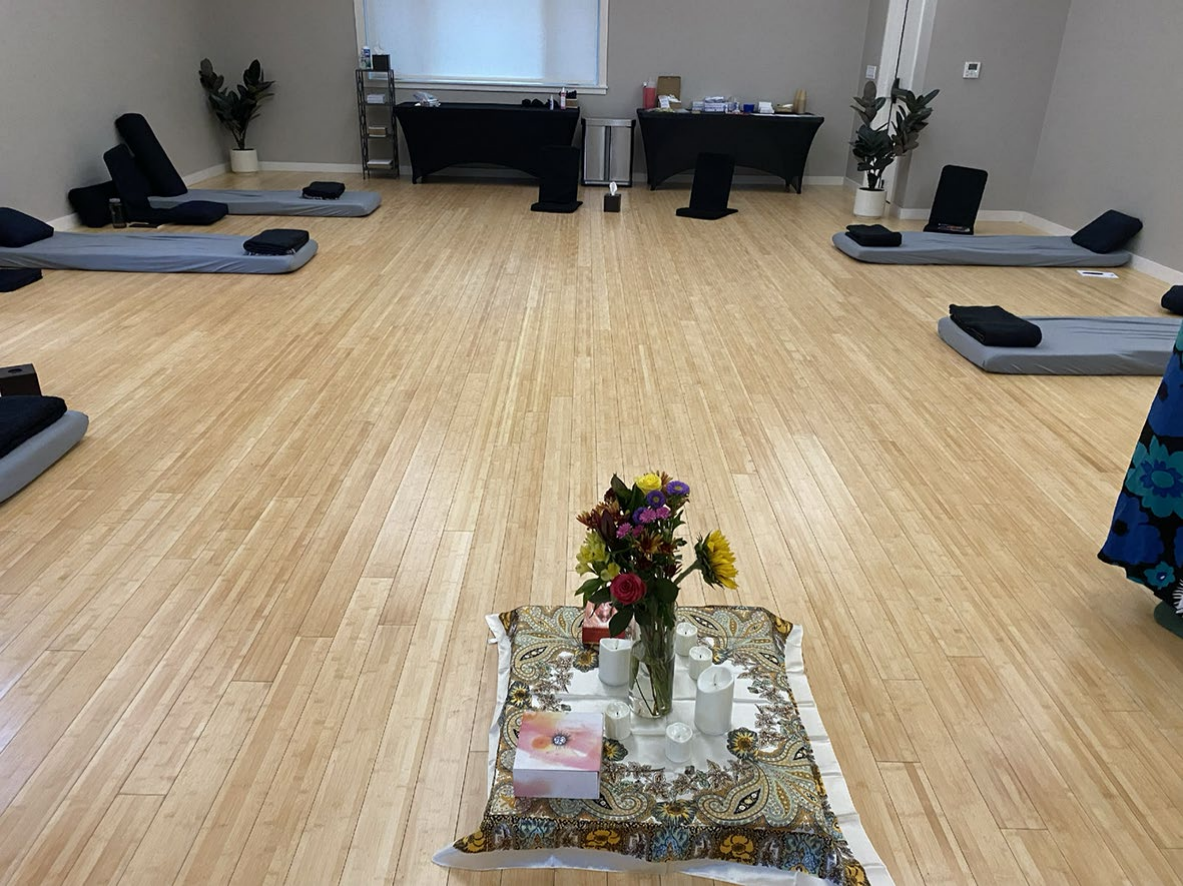
The altar in the training room. Photo by the Author

**Fig 4:**
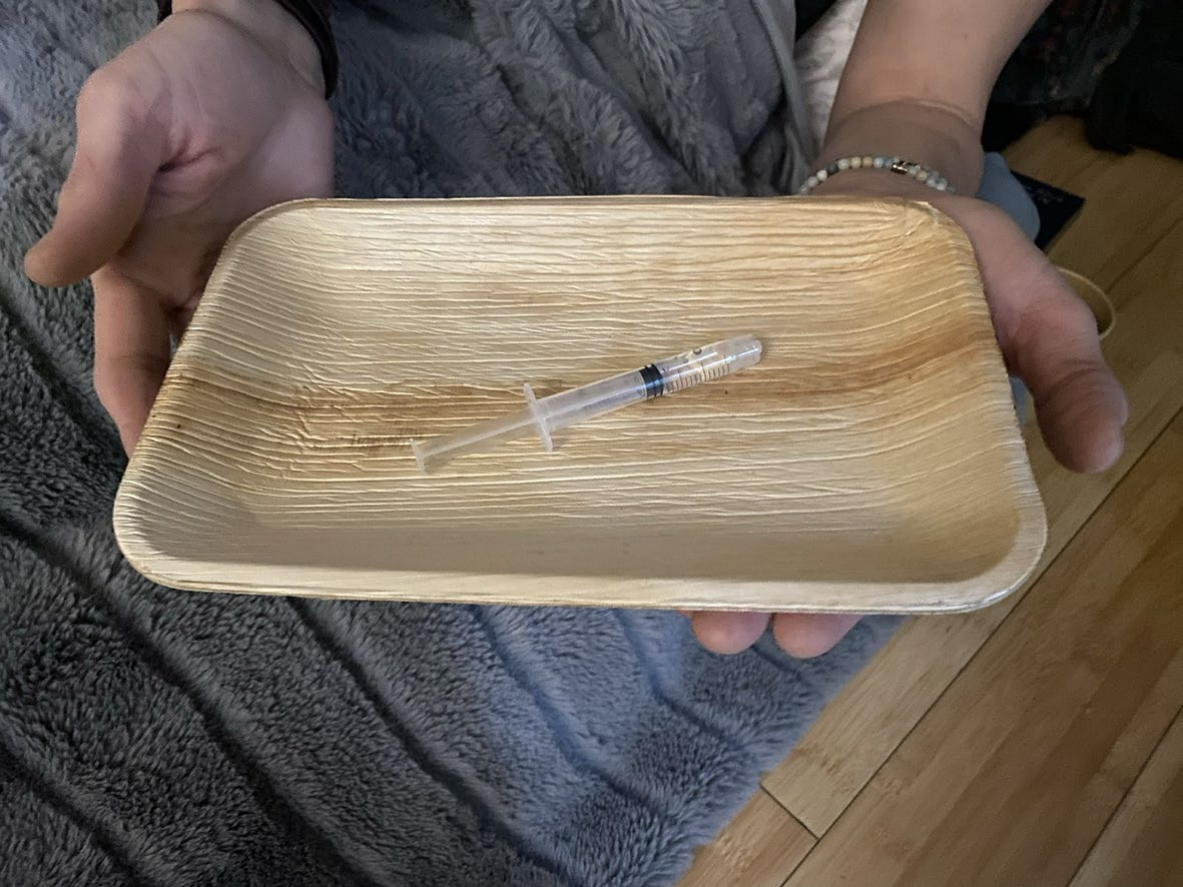
"Blessings on Your Journey." Photo by the Author

After lunch, we split into our small pod groups and find our training rooms. The space is organized around an altar that has been constructed at the center of the room and is decorated with a bouquet of fresh flowers, flickering battery-operated tea-lights, tarot cards, and whatever small objects the participants have chosen to place there. Soft mats and blankets are arranged in a circle around the altar. Eyeshades have been provided. Soon it's time for the dosing to begin. Everyone taking the medicine puts their name tag at the foot of their mat. The doctor hands out wooden trays with a syringe of liquid ketamine for oral administration to each person and says “blessings on your journey.” One of the instructors sings a prayer in another language and plays a wooden flute as she circles the altar. The participants squirt the ketamine into their mouths with the syringe and swish it around, holding it there for 15 minutes while the facilitators read poems like “The Guesthouse” by Rumi. After 15 minutes, they spit the ketamine into cups and lay down (Figs. 3, 4).

During dinner and breakfast, people talk about their ketamine experiences. A doctor and ketamine provider tells me about the time she took a low dose of intramuscular ketamine and became a vibrating insect inside the petals of a dahlia. She says in the clinic, sometimes “I pray to the vials for certain patients” but assures me she “only does woo with consent.”^11^ Anthropologists and scholars of science and technology have shown that science can sometimes incorporate religion in unexpected ways, especially when the concept of *assistance* may hold multiple meanings (Roberts, 2012). In her work on assisted reproduction in the Andes, Elizabeth Roberts describes how IVF laboratory biologists regularly prayed to God, made signs of the cross, or touched images of Jesus and the Virgin Mary which were affixed to lab equipment as they did important steps in the process of in-vitro fertilization (Roberts, 2012, 9). Lab clinicians saw their work as *assisted* by God, who would ultimately determine if the procedure was successful or if an embryo would implant. Such prayers in the laboratory challenge the secular modern assumption that reproduction, whether natural or assisted, is separate from spiritual domains (Roberts, 2012, 13). By invoking the presence of God in the laboratory, Roberts argues, Ecuadorian doctors sought to manage situations of extreme uncertainty.

Spiritual practices and materials were similarly present in the ketamine retreat, where the inability to predict the content and tone of one’s impending ketamine experience charged the air with uncertainty, anticipation, and anxiety. Through building altars, offering blessings, and reading tarot, the instructors worked to transform an anesthetic in a vial into a medicine with mystical power and significance. The sacred setting was further bolstered by a playlist which featured a selection of meditative New Age/ambient music that included introspective songs like “Following the Seasons” by Aisha Akebono and the spiritual looping mantras of “Storm of Prayers” by Shaman’s Dream.^12^ Such sounds and practices both shaped the experience of ketamine and were inspired by the phenomenology of ketamine itself which, depending on the dose, transported people into non-ordinary states of consciousness where they accessed unconscious material and repressed memories, experienced their own death, became non-human objects, and were confronted with existential meaning. When faced with the possibility of undergoing such a wide range of extreme, existentially significant experiences in a clinical setting, the presence of blessings and spiritual material did not seem unwarranted. As Palitsky et al. have pointed out, “68% to 86% of individuals who have taken psychedelics in a therapeutic setting reported the experience as among the most spiritually meaningful in their lives” (Palitsky et al., 2023, E2).

On the final day of the retreat, we practice with high-dose intramuscular ketamine. The set-up is similar from the day before, with the altar, the opening prayer, the offering of ketamine on a plate, and the doctor wishing each participant “blessings on your journey” after injecting ketamine into arms. This time people have stronger reactions. One person says she went under the earth and became a cave, another saw jack-in-the-box faces and then became a piece of thread in tweed fabric, one man met God and shouted in ecstasy, while another experienced his own death. “When you experience your own death and come back––what ICD code is that?” he asked after the session, referencing the International Classification of Diseases in his comment on the impossibility of fitting ketamine into the biomedical paradigm without medicalizing spiritual encounters. As we close the ceremony, the doctor tells us that “the veil is very thin,” and reminds us to journal to “keep the portals open” and to stay in a sacred space for as long as we can so we can continue to remember the insights we’ve been given. In the ketamine-assisted psychotherapy training, disaffected psychiatrists transformed a surgical anesthetic into a spiritual substance capable of weakening the boundary between material and spiritual worlds. Such embodied practices of secular mysticism create space for spiritual experience within a medical field dominated by a biological disease model of mental illness.

## Science in the Church

While psychiatrists worked to make biomedicine mystical in the ketamine retreat, in a nearby entheogenic church, members actively sought to incorporate science into their sacred practice. In the Zoom room of the Sanctuary of Infinite Bloom people connect from their homes in California, Washington, Oregon, and beyond to join the weekly Satsang, a Sanskrit term referring to a form of devotional gathering. Zoom boxes reveal a group of mostly white men and women spanning multiple generations. One member sits cross-legged outside with a blanket wrapped around his shoulders, plants blowing in the breeze behind him. Another has projected his own surreal background of psychedelic snakeskin. However, most of the participants have connected from rather ordinary looking domestic spaces of kitchens, living rooms, and bedrooms. A new member introduces himself to the group and says he has joined because he wants to learn about psychedelics and “how they help to heal and change our perspective.”

As John, the charismatic pastor, opens the service, he begins by explaining that the church is built around the idea of “least dogma.” “Least dogma” refers to the radical openness of this self-described “postmodern” congregation which actively welcomes people from all religions, faiths, wisdom lineages, and political orientations and promotes a vision of religion as a non-dogmatic, individual, and personal practice. The concept of “least dogma” resonated with church members, the vast majority of whom described themselves in interviews as “spiritual but not religious,” often criticizing what they perceived to be the “dogmatic” nature of religion. In this newly formed, multi-sacrament church, there is only one core belief required of its members: that they are “open to the possibility that, engaged through careful and respectful practice, entheogens can connect us to a direct experience of the divine, within this lifetime.”

The weekly Satsang is a virtual space where members of the Sanctuary come together to share their experiences with psychedelic sacraments, build community, and learn about sacred plants and molecules. It is not uncommon to hear references to William James, Ram Das, Aldous Huxley, Habermas, or María Sabina. Everyone’s favorite time is “Plant Time,” a part of the service that involves PowerPoint lectures on the indigenous history, chemical make-up, identification, preparation, and consumption of entheogenic plants and molecules. During Plant Time, Pastor John nimbly tacks back and forth across multiple ontologies while handling cacti, seeds, saplings, and fungi from his garden for the viewers on the screen––showing members, for example, how to cut, dry, and process *huachuma,* while emphasizing that, depending on your cultural background and belief system, it contains either a spirit or a chemical molecule that binds to serotonin 5-HT-2A receptors in the brain, or both.

Since the 2018 publication of Michael Pollan’s bestselling book on psychedelics, *How to Change Your Mind,* and the decriminalization of entheogenic plants in cities across the US, an interest in psychedelics as tools for healing and self-transformation has quickly grown among the American public.^13^ While people were drawn to the church for a range of reasons, a dominant refrain was the desire to use psychedelics for healing trauma, personal growth, and spiritual development in a legal setting, often after embarking on “journey work” either on their own or with underground therapists and guides. As one white professional in his 50s explained during an interview, “I really do believe that these are sacred substances” before adding, “I don't really want to do 'em with someone in a white coat in some clinic somewhere…and along with that is…I prefer not to go to jail. And at this point, the only way you can use these substances is either with a doctor or in a sincerely religious context.”

Although psychedelics like ayahuasca, psilocybin, mescaline, and 5-MEO-DMT are Schedule 1 substances in the United States, the Religious Freedom Restoration Act (RFRA), provides the possibility of religious protection to groups like this that use psychedelics as sacraments, as long as they can prove they are a central element of sincere religious practice. Today, outside of clinical trials, ketamine clinics, and licensed psilocybin therapists and facilitators in Oregon, Colorado, and New Mexico, entheogenic churches with DEA approval are the only spaces where psychedelic substances can be used legally in the United States.

As a self-described “science-positive, postmodern” church, the domains of science and religion frequently blend together at the Sanctuary of Infinite Bloom. This entanglement appears not only in the discourse of Least Dogma that allows for multiple epistemologies and ontologies to coexist, but also throughout regular church gatherings. During a Satsang presentation on “helper plants,” Moon, a church member, began playing a recording of an Amazonian ayahuasca *icaro* about curing the body and cleansing the spirit (*cura cura cuerpicito, limpia, limpia espiritito…*) before launching into a lecture on the chemistry and psychopharmacology of the bobinsana plant (*calliandra angustifolia*). Moon showed slides with images of chemical diagrams while talking about harmala alkaloids (monoamine oxidase inhibitors), explaining that the plant opens up a channel to GABA receptors in the brain. Moon says he wants to do a deep dive into the neurobiology because he “likes the mechanistic understanding.”

**Fig. 5:**
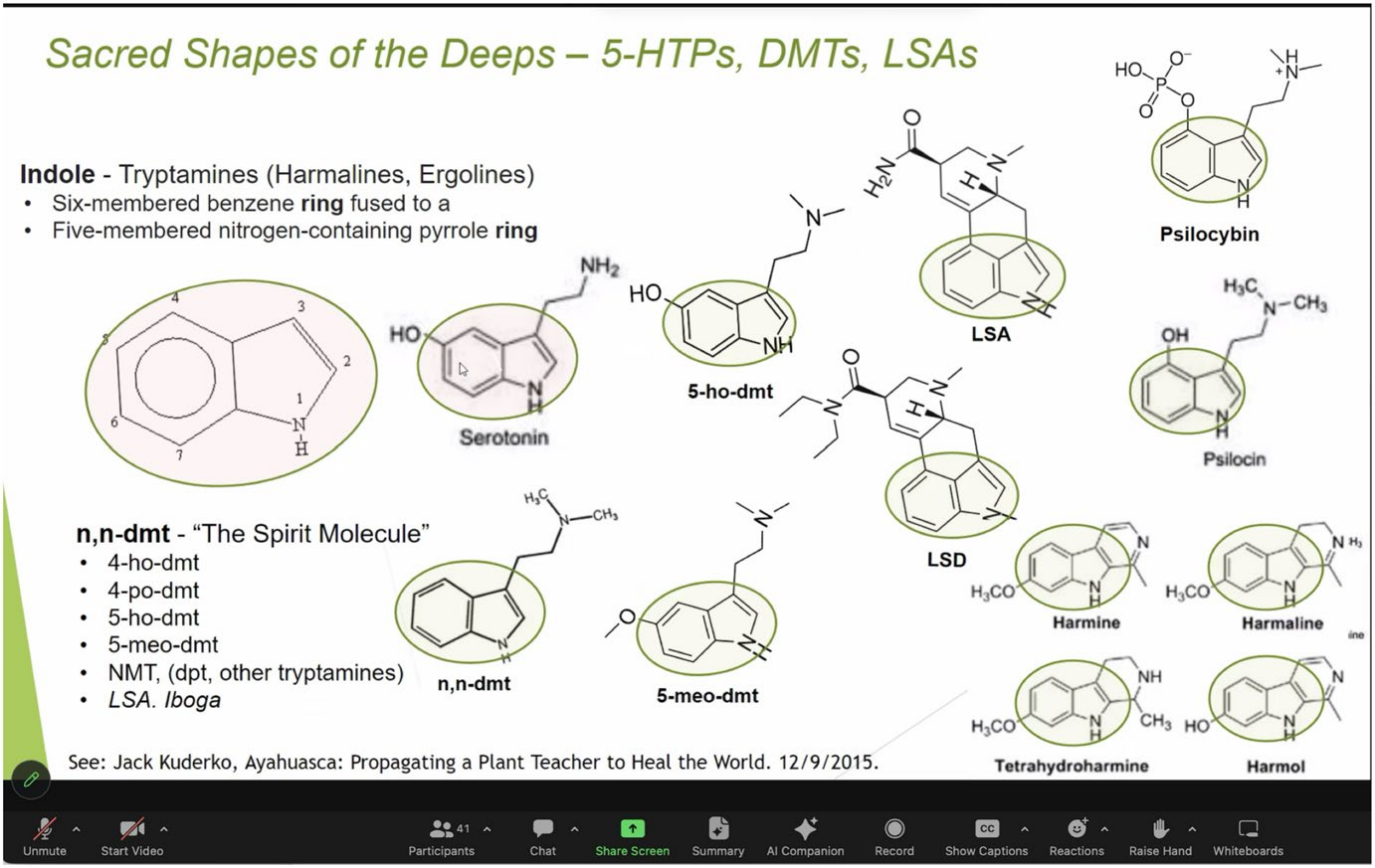
Chemistry slide on “Sacred Shapes of the Deeps” shown during a church Satsang. Photo by the Author

Powerpoint presentations at the Sanctuary seamlessly juxtapose slides on Indigenous use, scientific names, and chemistry illustrating the “sacred geometry” of tryptamines like DMT also known as the “spirit molecule” due to its regular ability to induce mystical near-death experiences (Strassman, 2001). One day while presenting, Pastor John commented on ontological controversies around psychedelics, and how “someone from the Sierra Madre Mazateca might say, ‘Pastor John what are you talking about, these mushrooms are beings?!’” His answer: “From my language and lineage it’s not so binary, Indigenous vs. Western…maybe we can learn from each other.” However, despite an attitude of epistemic and ontological openness, knowledge of chemistry plays an especially important role in the church. Pastor John often says that the “indole shape is like what a cross would be for a Christian.” An “indole” is an organic chemical structure made of a six-membered benzene ring connected to a 5-membered nitrogen-containing ring. Because serotonin contains an indole ring, psychedelics containing the indole shape, such as LSD, psilocybin, and DMT, are able to bind to the 5-HT-2A receptors in the brain. From a scientific perspective, indoles are the chemical structure that make classical psychedelic experience possible. The indole ring is the core chemical scaffold for several of the sacraments of the Sanctuary of Infinite Bloom (Fig. 5).

Pastor John’s emphasis on a divine chemistry resonated with church members, a number of whom were scientists and doctors who maintained faith in empiricism and the materialist, scientific perspective while also seeking spiritual experience. During an ordination ceremony, a new minister spoke about her struggle to balance her identity as a scientist with her decision to join the church. “It’s hard for me, a secular scientist and atheist, to accept the idea of a church,” she explained, audibly gagging after the word “church.” “So, I was so delighted to hear Pastor John talk about Least Dogma. The only thing I really believe in is possibility. So here I am, two years later, ready to bring a direct experience of the divine to others in the church through sacred plant medicine.” The entheogenic church offers a unique approach to the secular problem of mysticism. Here church members respond to the challenge of “secular doubt,” in which the existence of the supernatural is not taken for granted (Luhrmann, 2012, 377), by welcoming the co-existence of multiple ontologies such that psychedelics can be both chemical compounds and more-than-human bearers of sacred knowledge. As Pastor John put it, in the church “it’s not either/or, it’s both/and.”

## Secular Mysticism

In his article “The Secular Body,” Charles Hirschkind writes that “the secular is the water we swim in” (Hirschkind, 2011, 634). What he means is that this dimension of modern experience is so ubiquitous that it is often difficult to notice it, let alone gain enough distance to analyze the role it plays in shaping social life. In this article, I’ve drawn attention to the ways a culture of secularity in the US that disavows the possibility of spirits in favor of materialist explanations is contending with the incorporation of mystical experiences into society via the mainstreaming of psychedelics. In the laboratory, mysticism is quantified and operationalized. Here, spirituality is tolerated as an object of empirical investigation and measurement or as an element of participant experience, as long as it does not transgress the scientific virtue of objectivity. The ketamine training illustrates the opposite response to the problem of mysticism. There, practicum participants are taught to critique the “Western biomedical paradigm” by rejecting scientific approaches to mental illness that disavow spiritual healing as instructors worked to transform biomedicine into a sacred substance. The entheogenic church offers a novel solution to the secular discomfort with mystical experience. By taking a post-secular stance (Habermas, 2008), the church developed its own form of secular mysticism in which science is not placed in opposition to religion, but is celebrated as one of many, co-existing epistemic truths. Frameworks like “Least Dogma” resonated with non-religious church members who yearned for a “direct experience of the divine” without renouncing their faith in scientific explanation.

This is a story about the problem of mysticism in a secular age (Taylor, 2007) as it plays out in the tension between the two sides of psychedelics: one medical and the other spiritual. The intensity of debates around the role of spirituality in psychedelic medicine speaks to the force of its presence both in the clinic and beyond. This will continue to be an issue, as long as psychedelic substances reliably occasion mystical experiences of dissolution, union, death, terror, revelation, and existential reflection.^14^ Such extraordinary states of consciousness demand engagement with spirituality, as people struggle to find meaning and make sense of what they’ve undergone. Mystical experiences, whether they take place in a clinic, laboratory, or church, often prove to be so powerful that they shake people out of secular ways of being-in-the-world, transforming the worldview of even the most ardent scientist-atheist into a spiritual believer.

At the same time, psychedelic mystical experiences are not a response to the substance alone, but are also a testament to the set and setting of secular malaise in the US. The purposeful spiritual enrichment of ketamine clinics, therapy offices, entheogenic churches, and even laboratories across the country illuminates an underlying yearning for a form of healing that moves beyond the dominant culture of Western biomedicine by offering existential meaning, experiences of transcendence, and a sense of enchantment. In a society marked by increased disasters, a mental health crisis, polarized politics, mass movements for racial justice, and declining faith in science, psychedelics are touted as utopian substances capable of “rewiring” default mode networks and generating more altruistic, liberatory, and interconnected ways of relating to self, others, and the environment (Forstmann & Sagioglou, 2017; Griffiths et al., 2018; Kettner et al., 2019; Lyons & Carhart-Harris, 2018; MacLean et al., 2011).^15^ In this way, the mainstreaming of mysticism via the psychedelic renaissance is comparable to other historical periods of crisis when people have turned to sacred plants, altered states, and a more holistic science as an answer to threats of cultural annihilation, catastrophe, nihilism, and disenchantment (Harrington, 1996; LaBarre, 1970).

Lastly, the desire to incorporate spiritual elements into psychedelic-assisted therapy indicates a broader dissatisfaction with North American psychiatry that, for the past 50 years, has been largely concerned with discovering the biological basis of mental illness over understanding the meaning of existential distress (Aviv, 2019; Frances, 2013; Gardner & Kleinman, 2019; Harrington, 2019; Hyman, 2012; Luhrmann, 2000; Morehead, 2021; Whitaker, 2010). As psychedelics and the mystical experiences they occasion enter mainstream medicine via psychiatry, they challenge the assumption that psychiatric disorders should be treated as medical conditions, as opposed to spiritual or existential afflictions. While some have conceptualized psychotherapy as spiritual practice (Corbett, 2011; Jung, 1969), this perspective has not been part of the dominant biological and behavioral paradigms in American mental health care which seek to treat brain disorders through chemical intervention or interrupt negative thought patterns with behavior modification. As one psychedelic-therapist-in-training put it, “psychedelics are challenging the world of health to realize that spiritual healing is part of that, but Western psychiatry doesn’t allow for it.” This critique speaks to the emergence of a broader cultural shift, in which trauma, psychiatry, science, religion, and psychedelics are coming together in a new historical formation where the boundaries between therapy and spiritual experience are beginning break down. The popularization of substances that occupy the border between medicine and sacrament ultimately reveals a society in search of novel forms of healing that might allow for a braiding of science and religion, the mystical and the therapeutic.

## Data Availability

No datasets were generated or analyzed during the current study.

## Footnotes

1 Schedule I substances are defined as “drugs with no currently accepted medical use and a high potential for abuse” (DEA, n.d.).

2 To see an image of the painting, “Stanislav Grof M.D., Cartographer of Consciousness,” visit: https://www.alexgrey.com/art/psychedelic/stanislav-grof-md-cartographer-of-consciousness

3 A “psychonaut” (i.e., “navigator of the psyche”) is a person who purposefully induces altered states of consciousness (through meditation, psychedelic substances, sensory deprivation, etc.) as a method to explore their own psyche.

4 See, for example, the recently published study: “Effects of Psilocybin on Religious and Spiritual Attitudes and Behaviors in Clergy from Various Major World Religions” (Griffiths et al., 2025).

5 As Abou Farman (2019, 59) puts it, the secular involves a set of assumptions about the world and the nature of reality, that “ground secularism as a project and make secular subjects.”

6 For a history of the use of psychedelics as weapons of mind-control during the cold war, see (Breen 2024).

7 However, such neoshamanic ayahuasca spirituality is often bolstered by primitivist fantasies of return to a premodern, exotic, and sacred past (Fotiou 2016).

8 Through historical readings of the biographies of key proponents of the disenchantment narrative, Josephson-Storm demonstrates how many of these figures, including Max Weber himself, were participating in their own experiences of seances and mysticism as news articles reported on the growth of occultist religious movements.

9 Although neuroscientists like David Olson have challenged the idea that the subjective effects of psychedelics are necessary for therapeutic efficacy, even Olson acknowledges the influence of this theory when he writes that “conventional wisdom holds that peak mystical experiences induced by psychedelics are a critical component of their therapeutic mechanisms of action” (Olson 2021, 563).

10 All names are pseudonyms.

11 “Woo” or “woo-woo” is a popular term referring to beliefs lacking scientific basis, often relating to mysticism, spirituality, and alternative medicine.

12 For a discussion of psychedelic playlists as the “hidden therapist,” see (Kaelen et al., 2018).

13 As an interview with Michael Pollan in Time Magazine notes, “Few people have done more to return psychedelics to the popular imagination—while lending them a dose of credibility—than author Michael Pollan” (Law 2022). See also Noorani (2020) on “the Pollan effect.”

14 Following from this, we might read the new development of “tripless psychedelics,” also known as “psychoplastogens,” as reflecting the secular desire to escape the problem of mystical experience altogether.

15 For a critical reading of the claim that psychedelics generate prosocial behaviors, see: (Langlitz 2020; Pace and Devenot 2021; Saldanha 2007).
